# A systematic review and meta-analysis of pregnancy and COVID-19: Signs and symptoms, laboratory tests, and perinatal outcomes

**DOI:** 10.18502/ijrm.v18i12.8022

**Published:** 2020-12-21

**Authors:** Soheil Hassanipour, Saeed Bagheri Faradonbeh, Khalil Momeni, Zahra Heidarifard, Mohammad-Javad Khosousi, Leila Khosousi, Hosein Ameri, Morteza Arab-Zozani

**Affiliations:** ^1^Gastrointestinal and Liver Diseases Research Center, Guilan University of Medical Sciences, Rasht, Iran.; ^2^Health Management and Economics Research Center, Iran University of Medical Sciences, Tehran, Iran.; ^3^Department of Public Health, Faculty of Health, Ilam University of Medical Sciences, Ilam, Iran.; ^4^Tehran University of Medical Sciences, Tehran, Iran.; ^5^Reproductive Health Research Center, Guilan University of Medical Sciences, Rasht, Iran.; ^6^Health Policy and Management Research Center, Department of Health Services Management, School of Public Health, Shahid Sadoughi University of Medical Sciences, Yazd, Iran.; ^7^Social Determinants of Health Research Center, Birjand University of Medical Sciences, Birjand, Iran.

**Keywords:** COVID-19, Pregnancy, Diagnosis, Signs and symptoms, Meta-analysis.

## Abstract

**Background:**

Coronavirus Disease 2019 (COVID-19) caused by severe acute respiratory syndrome coronavirus 2 appeared in December 2019 in Wuhan, China.

**Objective:**

To investigate the clinical manifestations including signs and symptoms, laboratory results, and perinatal outcomes in pregnant women with COVID-19.

**Materials and Methods:**

Scholarly databases such as PubMed via LitCovid hub, Embase, Scopus, Web of sciences, and Google scholar were searched on April 7, 2020. Meta-analysis was performed via comprehensive meta-analysis software using the Mantel-Haenszel method. The event rate with 95% CI was calculated for each variable.

**Results:**

Ten studies were selected. The pooled prevalence for fever, post-partum fever, cough, myalgia, fatigue, dyspnea, sore throat, and diarrhea were 66.8%, 37.1%, 35%, 24.6 %, 14.9%, 14.6%, 11.5%, and 7.6%, respectively. Laboratory test results were 49.8% for lymphopenia, 47.7% for leukocytosis, 83.7% for elevated neutrophil ratio, 57% for elevated C-reactive protein, and 71.4% for decreased lymphocyte ratio. The rate of cesarean section for delivery in all cases was 84%. Of the newborns of the corona-positive mothers, only one had a positive test result. Also, there was only one death due to a decreased lymphocyte ratio.

**Conclusion:**

Fever was the most common sign and symptom in pregnant women with COVID-19. Among the laboratory tests, the highest amount was related to elevated neutrophil ratio. It seems that due to the differences between pregnant women and the general population, special measures should be considered to treat these patients.

## 1. Introduction

Coronavirus Disease 2019 (COVID-19) caused by severe acute respiratory syndrome coronavirus 2 (SARS-CoV-2) appeared for the first time in December 2019 in Wuhan, China. Following that, the disease spread rapidly around the world to the point where it was confirmed a pandemic by the World Health Organization (WHO) (1, 2). COVID-19 is an infectious disease with respiratory symptoms almost similar to SARS (2003) and MERS (2012) epidemics (3, 4). In some cases, the disease can lead to a sensitive respiratory condition, many of which require specialized management in the intensive care unit (ICU) (5).

Moreover, respiratory droplets along with close contact transmission are the considerable routes of transmission. Aerosol transmission is also possible in a close environment when exposed to high concentrations of aerosol for a protracted period (6). On the other hand, touching surfaces or objects that are touched by an infected person can also transmit the disease (7). A study has also shown that older age and comorbidity play an important role in determining the severity and clinical consequences of the disease (8).

Because most studies have focused on patients infected with the new coronavirus in the general population, bounded details are available regarding pregnancy outcomes in women infected with COVID-19. It has caused particular concern among pregnant women, as both SARS-CoV and MERS-CoV viruses have been shown to cause severe side effects in pregnant women (9, 10). In 2004, Wong and colleagues conducted a study on pregnant women with SARS in Hong Kong and observed that the pregnant women showed higher rates of death and mortality (11). Similarly, a study by Mertz and colleague showed that women infected with influenza were at a higher risk than healthy pregnant women (12). Chen and co-authors also reported that pregnancy with pneumonia could be associated with the risk of cesarean delivery, preterm delivery, a decrease in the baby's Apgar score, weight loss at birth, etc. (13).

It is obvious that a parturient woman has a relatively depressed immunity or immune suppression, and in theory, they could be more at risk of contracting the virus. Also, confronting the SARS-CoV-2 during pregnancy is a serious threat to pregnant women and their fetuses (14, 15). Therefore, it is pertinent to prevent pregnant women from being infected during the epidemic/pandemic period such as that of COVID-19, a disease without an approved treatment.

Pregnant women are at a risk of infection to respiratory pathogens and severe pneumonia because they are in an immunosuppressive state and changes in physiological adaptation during pregnancy (e.g., increased diaphragm levels, increased oxygen consumption) can cause hypoxia intolerance in such patients. For instance, the outbreak of influenza in 1918 caused a total mortality of 2.6% in the population, however in pregnant women, it was about 37% (16). Additionally, pregnant women were also observed to have a higher risk of complications from the H1N1 epidemic influenza virus infection in 2009 and were hospitalized fourfold more than the other patients (relative risk 4.3 95% CI: 2.3-7.8) (17). Therefore, it is important to study the signs and symptoms of COVID-19 in pregnant women as understanding the disease and its effects on newborns are very important.

Thus, this study is aimed at investigating the clinical manifestation including the signs and symptoms, laboratory results, and prenatal outcomes in pregnant women with COVID-19.

## 2. Materials and Methods

This systematic review and meta-analysis was followed by the Preferred Reporting Items for Systematic Reviews and Meta-Analysis (PRISMA) statement (18).

### Eligibility criteria

All included studies were investigated COVID-19 in pregnant women or during pregnancy and were in the English language. Studies were excluded if the researchers didn't have access to the full-text of the article or the data about the outcomes were not sufficient. Also, studies that were not peer reviewed were excluded.

### Information sources and search

Scholarly databases including PubMed via LitCovid hub, Embase, Scopus, Web of Sciences, and Google Scholar were searched using specific keywords (“2019 nCoV" OR 2019nCoV OR “2019 novel coronavirus" OR “COVID 19" OR COVID19 OR “new coronavirus" OR “novel coronavirus" OR “SARS CoV-2" OR (Wuhan AND coronavirus) OR “COVID 19" OR “SARS-CoV" OR “2019-nCoV" OR “SARS-CoV-2" AND pregnancy OR “pregnant women”) on April 7, 2020. Our search was not limited by the type of study or publication date but by the studies with full-text in the English language. We also searched the references of the included studies for capturing potential studies in the field. For incomplete data, the corresponding author of the article was contacted for more information.

### Study selection

After importing the records to EndNote X7, the duplicate records were removed and then screened based on the title, abstract, and full-text considering the eligibility criteria. All stages were conducted using two independent reviewers and the potential disagreements were solved through consultation with a third reviewer.

### Quality appraisal

Two independent reviewers assessed the included studies for quality issues. Because the final studies were case-series and case-control, the JBI checklists related to this type of study were used. These checklists include 10 questions for case-control and case-series studies. These questions investigate issue regarding domain such as inclusion criteria, reliability and validity of methods, sampling process, transparency in data and results, and statistical analysis. The detail about each question has been mentioned at the end of the questionnaire (Supplementary 1). We scored one for yes and zero for no in each question (19, 20).

### Outcomes measures

The investigated outcomes were signs and symptoms (cough, diarrhea, dyspnea, fatigue, fever, myalgia, sore throat, and post-partum fever), laboratory test results (lymphopenia, leukocytosis, elevated neutrophil ratio, elevated C-reactive protein, and decreased lymphocyte ration), type of delivery (cesarean), and perinatal outcomes (COVID-19 positive, low birth weight, premature, complication, and death). For all outcome variables, we extracted the number of events and sample size.

### Data analyses

Meta-analysis was performed for the signs and symptoms, laboratory tests, and type of delivery using the event rate (the proportion of the occurrence of an event in the subjects to the total subjects under study) with CMA (version 2) software using the Mantel-Haenszel method. In addition, narrative synthesis was used for reporting the results of the perinatal outcome. The Q-value was applied to discover between-study heterogeneity, and *I* ${}^{2}$ values were calculated to assess statistical heterogeneity. Random-effect model was used based on the level of heterogeneity. Based on the Cochrane criteria, we used the random-effect model when the heterogeneity was over 50% (21). The event rate with 95% CI was calculated for each variable. Egger's test and visual inspection of the funnel plot were used for assessing publication bias. In addition, a meta-regression was conducted for an association between the mean age and each sign and symptoms, laboratory test, and type of delivery.

## 3. Results

### Description of search

**Figure 1:**
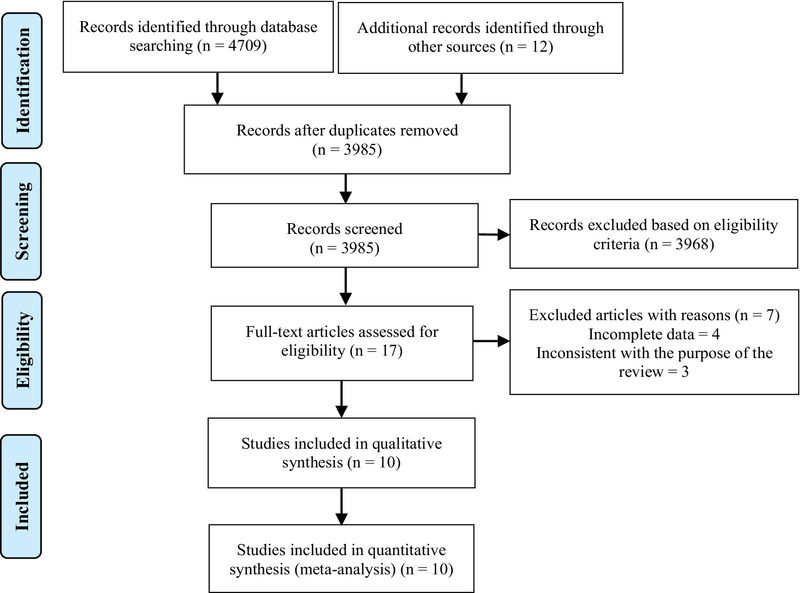
Study selection flow diagram.

After searching all international databases, 4,721 articles were found; after removing the duplicate articles, 3,985 articles were examined in terms of title and abstract, of which 17 articles were passed to the next stage. Finally, after reviewing the full texts of the articles, 10 articles entered the systematic review (22-31). In the screening stages of studies, they were excluded for a variety of reasons, which included unrelated topics (two articles), unassociated population (four articles), and duplicate study (one article). The overall sample size of the included studies was 135 pregnant women diagnosed with COVID-19 (Figure 1).

### Characteristics of included studies

Based on the geographical location, all included studies were performed in China. Table I shows the summary characteristics of the included studies.

### Quality assessment

Based on the results of the quality assessment, seven studies were good quality and three were average (Table II).

### Heterogeneity

Based on the data analysis, a high level of heterogeneity was not observed in the findings. In some cases with high heterogeneity, the random effect was used (Table III).

### Synthesis of results

#### Signs and symptoms

**Figure 2:**
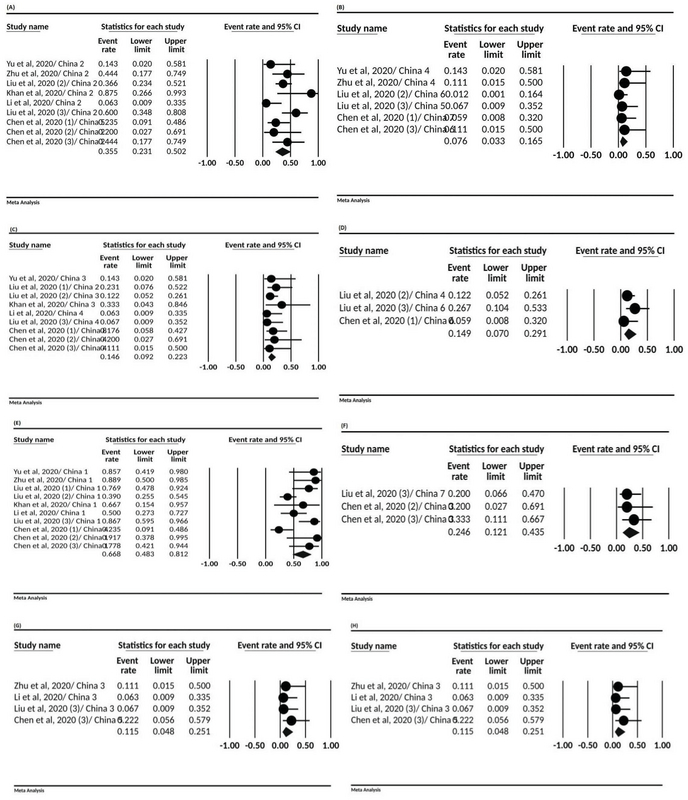
The forest plot presenting event rate and 95% CI for the signs and symptoms in pregnant women with COVID-19; (A) cough, (B) diarrhea, (C) dyspnea, (D) fatigue, (E) fever, (F) myalgia, (G) post-partum fever, and (H) sore throat.

Various signs and symptoms have been reported in studies. Of these, the highest was fever with 66.8% (95% CI; 48.3-81.2). Other reported signs and symptoms were: post-partum fever (37.1%, 95% CI; 18.5-60.6), cough (35.5.9%, 95% CI; 23.1-50.2), myalgia (24.6%, 95% CI; 12.1-43.5), fatigue (14.9%, 95% CI; 7-29.1), dyspnea (14.6%, 95% CI; 9.2-22.3), sore throat (11.5%, 95% CI; 4.8-25.1), and diarrhea (7.6%, 95% CI; 3.3-16.5) (Figure 2, Table III).

#### Laboratory tests

**Figure 3:**
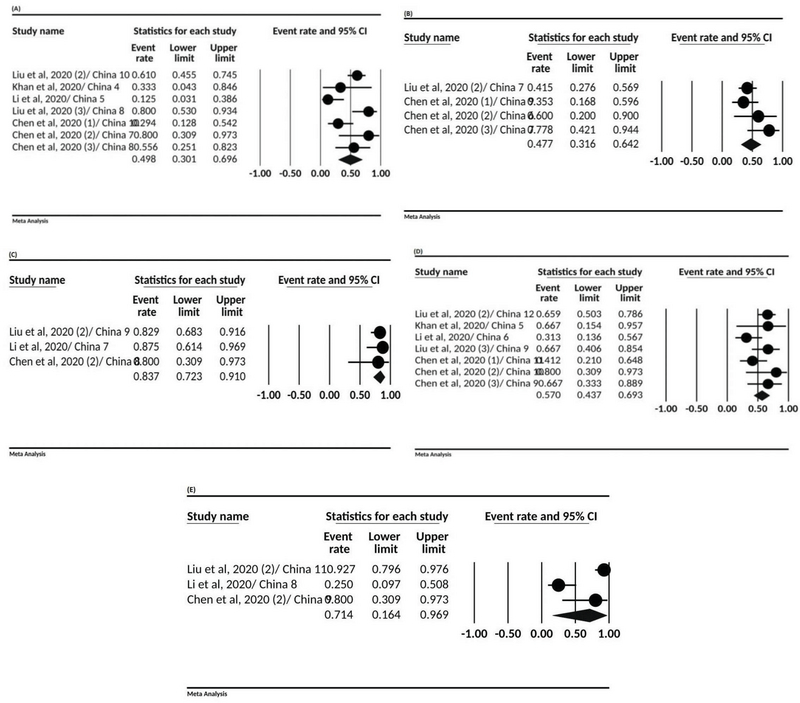
The forest plot presenting event rate and 95% CI for the laboratory tests in pregnant women with COVID-19; (A) lymphopenia, (B) leukocytosis, (C) elevated neutrophil ratio, (D) elevated C-reactive protein, and (E) and decreased lymphocyte ration.

Based on data analysis, lymphopenia with 49.8% (95% CI; 30.1-69.6), leukocytosis 47.7% (95% CI; 31.6-64.2), elevated neutrophil ratio 83.7% (95% CI; 72.3-91.0), elevated C-reactive protein 57% (95% CI, 43.7-69.3), and decreased lymphocyte ratio 71.4% (95% CI; 16.4-96.9) were observed in the studies (Figure 3,Table III).

#### Type of delivery

**Figure 4:**
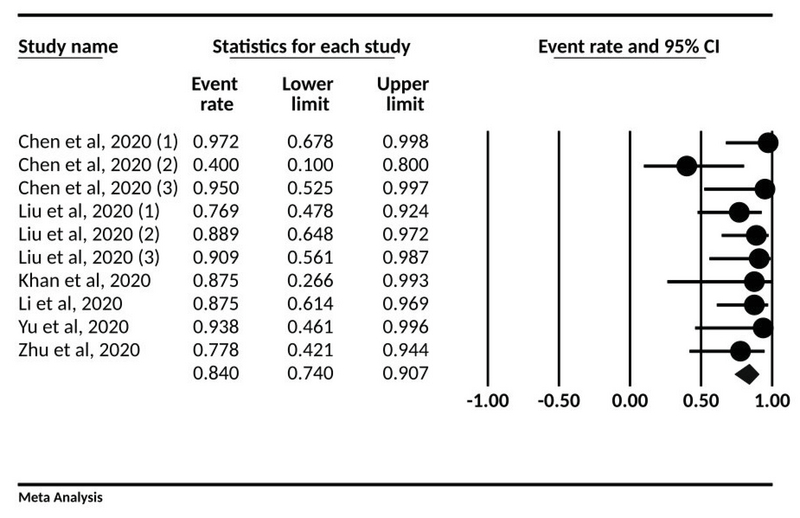
The forest plot presenting event rate and 95% CI for the type of delivery in pregnant women with COVID-19.

According to the results, the rate of cesarean section for delivery in all cases was 84% (95% CI; 74-90.7) (Figure 4, Table III).

#### Perinatal outcomes

According to the results, of the newborns of the corona-positive mothers, only one had a positive test result. Also, there was only one death due to DIC (Table IV).

### Results of meta-regression

According to the findings, the only factor that could be examined in this section was the mean age of pregnant women. Data analysis showed that older pregnant women have a significantly higher fever rate (Coefficient = 0.477, p = 0.033). For the type of delivery, the higher average age of pregnant women significantly associated with a higher rate in cesarean delivery (Coefficient = 0.433, p = 0.016) (Table V).

### Publication bias 

Visual inspection of funnel plot and Egger's tests did not indicate evidence of publication bias (p = 0.127).

**Table 1:** Basic information about the included studies


**Author, year (Ref)**	**Country**	**Setting**	**Time period**	**Study design**	**Sample size**	**Mean age (Year) (Range)**	**Mean gestational age (Week) (Range)**
**Chen ** ***et al.*** **, 2020-c (14)**	China	Renmin hospital	From January 30-February 23, 2020	Case series	17	29.5	<37 = 3 >37 = 14
**Chen ** ***et al.*** **, 2020-a (13)**	China	Maternal and child hospital of Hubei province	From January 20-February 10, 2020	Case series	5	28.4 (25-31)	38-40
**Chen ** ***et al.*** **, 2020-b (22)**	China	Zhongnan hospital	From January 20-January 31, 2020	Case series	9	32.5 (26-40)	36-39
**Khan ** ***et al.*** **, 2020 (25)**	China	Renmin hospital	From January 28-March 1, 2020	Case series	3	29.3 (27-33)	(34-39)
**Li ** ***et al.*** **, 2020 (24)**	China	Hubei provincial maternal and child health center	From January 24-February 29, 2020	Case-control	16	30.9	38
**Liu ** ***et al.*** **, 2020-c (28)**	China	Hospitals outside of Wuhan	From December 8, 2019-February 25, 2020	Case series	13	29.6 (22-36)	(25-38)
**Liu ** ***et al.*** **, 2020-b (26)**	China	Multicenter	From January 27-February 14, 2020	Case-control	41	30.5 (22-42)	NR
**Liu ** ***et al.*** **, 2020-a (27)**	China	Union hospital	From January 20, 2020-February 10, 2020	Case series	15	32 (23-40)	32 (12-38)
**Yu ** ***et al.*** **, 2020 (30)**	China	Tongji hospital	From January 1-February 8, 2020	Case series	7	32 (29-34 )	39 (37-41)
**Zhu ** ***et al.*** **, 2020 (31)**	China	Multicenter (five hospitals)	From January 20-February 5, 2020	Case series	9	29.5 (25-35)	(31-42)
NR: Not reported							

**Table 2:** JBI critical appraisal checklist applied to the included studies


**Author name/year (Ref) **		**Q1**	**Q2**	**Q3**	**Q4**	**Q5**	**Q6**	**Q7**	**Q8**	**Q9**	**Q10**	**Overall quality**
**Case series**												
	**Liu ** ***et al.*** **, 2020-a (27)**	Yes	Yes	Yes	Yes	Yes	No	Yes	N/A	No	Yes	7/10
	**Liu ** ***et al.*** **, 2020-c (28)**	Yes	Yes	Yes	Yes	N/A	Yes	Yes	Yes	N/A	Yes	8/10
	**Zhu ** ***et al.*** **, 2020 (31)**	Yes	Yes	Yes	Yes	No	Yes	Yes	Yes	No	Yes	8/10
	**Yu ** ***et al.*** **, 2020 (30)**	Yes	Yes	Yes	Yes	No	No	Yes	Yes	N/A	Yes	7/10
	**Khan ** ***et al.*** **, 2020 (25)**	Yes	Yes	N/A	Yes	No	Yes	N/A	No	No	Yes	5/10
	**Chen ** ***et al.*** **, 2020-a (13)**	Yes	Yes	N/A	Yes	No	Yes	N/A	Yes	No	Yes	6/10
	**Chen ** ***et al.*** **, 2020-b (22)**	Yes	Yes	Yes	Yes	Yes	N/A	N/A	Yes	No	Yes	7/10
	**Chen ** ***et al.*** **, 2020-c (14)**	Yes	Yes	Yes	Yes	No	No	Yes	Yes	No	Yes	7/10
**Case-control**												
	**Liu ** ***et al.*** **, 2020-b (26)**	Yes	Yes	Yes	Yes	Yes	Yes	N/A	Yes	Yes	No	8/10
	**Li ** ***et al.*** **, 2020 (24)**	Yes	Yes	Yes	Yes	No	Yes	N/A	N/A	Yes	No	6/10
**Case series design questions:** Q1. Were there clear criteria for inclusion in the case series? Q2. Was the condition measured in a standard, reliable way for all participants included in the case series? Q3. Were valid methods used for identification of the condition for all participants included in the case series? Q4. Did the case series have consecutive inclusion of participants? Q5. Did the case series have complete inclusion of participants? Q6. Was there clear reporting of the demographics of the participants in the study? Q7. Was there clear reporting of the clinical information of the participants? Q8. Were the outcomes or follow-up results of cases reported? Q9. Was there clear reporting of the presenting site(s)/clinic(s) demographic information? Q10. Was the statistical analysis appropriate? **Case-control design questions:** Q1. Were the groups comparable other than the presence of disease in cases of the absence of disease in controls? Q2. Were cases and controls matched appropriately? Q3. Were the same criteria used for the identification of cases and controls? Q4. Was exposure measured in a standard, valid, and reliable way? Q5. Was exposure measured in the same way for cases and controls? Q6. Were confounding factors identified? Q7. Were strategies to deal with confounding factors stated? Q8. Were outcomes assessed in a standard, valid, and reliable way for cases and controls? Q9. Was the exposure period of interest long enough to be meaningful? Q10. Was an appropriate statistical analysis used?												

**Table 3:** Results of heterogeneity among included studies


**Variable**	**Sub-groups**	**#No. of studies**	**Event rate (%) 95% CI**	**Q-value**	**Df (Q)**	**I${}^{2}$**	**P-value**	**Selected model**
**Signs and symptoms**	Cough	9	35.5, (23.1-50.2)	13.93	8	42.60	0.083	Random
	Diarrhea	6	7.6 (3.3-16.5)	2.65	5	0.0	0.754	Random
	Dyspnea	9	14.6 (9.2-22.3)	2.55	8	0.0	0.895	Random
	Fatigue	3	14.9 (7-29.1)	2.79	2	28.52	0.247	Random
	Fever	10	66.8 (48.3-81.2)	26.63	9	66.2	0.002	Random
	Myalgia	3	24.6 (12.1-43.5)	0.59	2	0.0	0.744	Random
	Sore throat	4	11.5(4.8-25.1)	1.71	3	0.0	0.633	Random
	Post-partum fever	4	37.1 (18.5-60.6)	6.01	3	60.1	0.016	Random
**Laboratory tests**	Lymphopenia	7	49.8 (30.1-69.6)	17.59	6	65.90	0.007	Random
	Leukocytosis	4	47.7 (31.6-64.2)	4.57	3	34.38	0.206	Random
	Elevated neutrophil ratio	3	83.7 (72.3-91.0)	0.236	2	0.0	0.889	Random
	Elevated C-reactive protein	7	57 (43.7-69.3)	8.89	6	32.53	0.180	Random
	Decreased lymphocyte ratio	3	71.4 (16.4-96.9)	19.47	2	89.73	<0.001	Random
**Type of delivery**	Cesarean	10	84 (74.0-90.7)	9.76	9	7.84	0.370	Random
NR: Not reported; CI: Confidence interval; DF (Q): Degrees of freedom (Cochran's Q); I${}^{2}$: I square								

**Table 4:** Perinatal outcomes of pregnant women with COVID-19


**Author, year (reference number)**	**Total sample size**	**Covid-19 Positive**	**Low birth weight**	**Premature**	**Complication**	**Outcome (Died)**
**Liu ** ***et al.*** **, 2020-a (27)**	13	0	NR	6	0	0
**Liu ** ***et al.*** **, 2020-b (26)**	NR	NR	NR	NR	NR	NR
**Liu ** ***et al.*** **, 2020-c (28)**	2	0	NR	0	0	0
**Zhu ** ***et al.*** **, 2020 (31)**	10	0	7	5	Multiple organ failure and DIC (1)	1
**Yu ** ***et al.*** **, 2020 (30)**	7	1	0	NR	0	0
**Khan ** ***et al.*** **, 2020 (25)**	3	0	0	1	0	0
**Li ** ***et al.*** **, 2020 (24)**	17	0	3	4	Intrauterine fetal distress (2)	0
**Chen ** ***et al.*** **, 2020-a (13)**	17	0	0	0	0	0
**Chen ** ***et al.*** **, 2020-b (22)**	5	0	0	0	0	0
**Chen ** ***et al.*** **, 2020-c (14)**	9	0	2	4	0	0
NR: Not reported						

**Table 5:** Result of meta-regression


**Variable**	**Sub-groups**	**Mean age**		
	**Coefficient**	**SE**	**P-value**
**Signs and symptoms**	Cough	0.192	0.207	0.354
	Diarrhea	0.103	0.392	0.792
	Dyspnea	-0.110	0.236	0.640
	Fatigue	0.676	0.405	0.095
	Fever	0.477	0.223	0.033
	Myalgia	0.0	0.322	0.999
	Sore throat	-0.117	0.520	0.821
	Post-partum fever	-0.001	0.192	0.995
**Laboratory tests**	Lymphopenia	0.309	0.252	0.220
	Leukocytosis	0.092	0.324	0.775
	Elevated neutrophil ratio	0.141	0.443	0.749
	Elevated C-reactive protein	0.057	0.231	0.802
	Decreased lymphocyte ratio	-0.649	0.431	0.131
**Type of delivery**	Cesarean	0.433	0.180	0.016
SE: Standard error				

## 4. Discussion

A total of 10 articles were reviewed in this study, which analyzed 135 pregnant women, all of whom were in the third trimester of pregnancy (22-31). These summary findings help healthcare workers better manage pregnant women with COVID-19, which could potentially reduce the side effects for women as well as their newborns.

The common clinical manifestations of pregnant women with COVID-19 include fever and cough, and the less common symptoms are sore throat and diarrhea. Postpartum fever is also more common in women after childbirth. However, the rate of fever in our study was lower than that of Guan and colleagues', who studied the symptoms of non-pregnant coronary artery disease and reported an 87.9% rate of fever. However, similar to our study, in their study, diarrhea was the least common (32).

In terms of laboratory demonstrations, elevated neutrophil ratio and decreased lymphocyte ratio are common. On the other hand, the prevalence of CRP elevated in our study was 57%. However, in Zhang and co-authors' study, this prevalence in a group of people with non-severe and severe patients was 88.9% and 96.4%, respectively (33). This indicates a more pronounced inflammation in patients with more severe conditions and given that pregnant women in this study were not in severe disease conditions, a lower percentage of increased CRP prevalence is justified. On the other hand, in Rodriguez-Morales co-workers study, the increased CRP prevalence was 58.3%, which is similar to our study (8). These differences in numbers can be explained due to the severity of the disease, and on the other hand, a more comprehensive examination is needed.

Lymphopenia and leukocytosis were less common in our study. However, in the study of Zhang and colleagues and Wang and colleague, which was performed on patients with COVID-19 (normal population), lymphopenia was the most common laboratory symptom and was 75.4% and 70.3%, respectively (33, 34). However, it should be noted that these numbers are a decrease in absolute lymphocyte count.

In our study, the majority of pregnancies ended up with cesarean section, which is much higher than the WHO's recommendation for vaginal route delivery (35), which can be determined by a gynecologist to prevent maternal respiratory distress during pregnancy.

In the current study, which examined 135 pregnant women with COVID-19 pneumonia, none of the patients with severe or dead pneumonia were infected with COVID-19 infection. Although SARS-CoV-2 has a common sequence with SARS of up to 85%, we need to be aware of the possibility that the course of the disease and the prognosis of this disease can follow the same SARS process in pregnant women (36, 37).

The current study does have some limitations. First, all patients registered in the included articles were in the third trimester of pregnancy, and the effect of the virus infection on the fetus in the first or second trimester was unknown. Second, due to the short duration of the outbreak, the long-term consequences of the disease on infants have not been possible and more studies are needed. Third, the low number of samples of articles included is another limitation of the work. Fourth, all included studies were from China.

## 5. Conclusion

In conclusion, pregnant women with COVID-19 pneumonia had diverse symptoms; however, fever and cough were the main clinical symptoms in those women. Although one infant was born with COVID-19 in the included studies, there was little evidence that COVID-19 was transmitted from mother to infant in late pregnancy. Therefore, the study of long-term outcomes on mother and child, as well as the vertical transfer of mother to child in second-trimester pregnancies and the first months after delivery, requires further studies.

##  Conflict of Interest

The authors declare no conflict of interest.
